# The Impact of COVID-19 Pandemic on the Academic Performance of Veterinary Medical Students

**DOI:** 10.3389/fvets.2020.594261

**Published:** 2020-10-06

**Authors:** Mohamed A. A. Mahdy

**Affiliations:** Department of Anatomy and Embryology, Faculty of Veterinary Medicine, South Valley University, Qena, Egypt

**Keywords:** academic performance, COVID-19, veterinary, online learning (OL), students, coronavirus

## Abstract

Many universities and colleges worldwide suspended classroom teaching due to the novel coronavirus pandemic and switched to online teaching. The current cross-sectional study was carried out to analyze the impact of COVID-19 lockdown on the academic performance of veterinary medical students and researchers. Veterinary medical students and researchers were invited to answer an online google form questionnaire. A total of 1,392 participants were from 92 different countries answered the questionnaire with response rate of 94.1%. The data showed that COVID-19 pandemic lockdown affected the academic performance of most participants (96.7%) with varying degrees. The mean evaluation score for the online education in general was 5.1 ± 2.4 while that for the practical parts was 3.6 ± 2.6. Although online education provides an opportunity for self-study, the main challenge that online education faces in veterinary medical science is how to give practical lessons. Since most of the subjects are practical; therefore, it is not easy to learn it online. Students think that it is difficult to fulfill the veterinary competencies only with online education system. Online education could be improved by making it more interactive, showing medical procedures in real situations, giving concise information, and providing 3D virtual tools to mimic the real situation.

## Introduction

Coronavirus disease 2019 (COVID-19) is firstly identified in Wuhan city, Hubei Province, China in December 2019 as a pneumonia of unknown origin (1). Later, the international committee on taxonomy of viruses (ICTV) identifies the causative agent of COVID-19 as a novel coronavirus, severe acute respiratory syndrome coronavirus−2 (SARS–CoV−2) (2). COVID-19 outbreak spreads rapidly not only in China, but also worldwide, therefore, the World Health Organization (WHO) has announced it as pandemic on March 12, 2020 (3). The total number of confirmed cases and mortalities are 23,491,520 and 809,970, respectively, in 216 countries as of August 25, 2020 (4).

Several governmental measures have been taken to counteract the risk of disease spreading. These measures include travel restrictions, mandatory quarantines for travelers, social distancing, bans on public gatherings, schools and universities closure, business closures, self-isolation, asking people to work at home, curfews, and lockdown (5, 6). Authorities in several countries worldwide have declared either lockdown or curfew as a measure to break the fast spread of virus infection (7). These measures have a negative worldwide effect on the business, education, health, and tourism (8).

COVID-19 pandemic has affected all levels of the education system (9). Educational institutions around the world (in 192 countries) have either temporarily closed or implemented localized closures affecting about 1.7 billion of student population worldwide (10). Many universities around the world either postponed or canceled all campus activities to minimize gatherings and hence decrease the transmission of virus. However, these measures lead to higher economical, medical, and social implications on both undergraduate and postgraduate communities (9, 11).

Due to the suspension of classroom teaching in many colleges and universities, a switch to the online teaching for undergraduate and graduate students becomes effective [reviewed in (12, 13)]. This form of learning provides an alternative way to minimize either the contact between students themselves or between the students and lecturers (8). However, many students have no access to the online teaching due to lack of either the means or the instruments due to economical and digital divide (14).

Few studies highlighted COVID-19 in relation to educational studies. COVID-19 has a profound impact on medical students, dental medical students, and radiology trainee (15–20). Recently, the American Veterinary Medical Association (AVMA) showed that COVID-19 adversely impacted veterinary practices based on a large survey inculing about 2,000 responses (21). However, there is no studies investigated the effect of COVID-19 on students in veterinary medical field. Therefore, the current study was conducted to analyze the impact of COVID-19 pandemic on the academic performance of veterinary medical students and researchers during the lockdown.

## Materials and Methods

### Questionnaire Design

An online anonymous questionnaire was designed and an initial test was done on 50 participants to ensure that the draft questionnaire was understandable. The aim and uses of data of the questionnaire were briefly explained at the beginning of the questionnaire. An online google form questionnaire (Supplementary File 1) link was shared with different veterinary groups in various social media platforms (Veterinary Facebook groups and Veterinary WhatsApp groups). Veterinary students and researchers were asked to answer the questionnaire for a research purpose. Participants were also asked to share the questionnaire link among their veterinary colleagues; therefore, the questionnaire could reach many participants. The final questionnaire for this study consisted of 18 questions (12 closed-ended and 6 open-ended) divided into two sections as follow: The first section included 8 questions about the demographic characteristics of participants (gender, age, country, residence place, university, program level, and academic year). The second section evaluated the effect of COVID-19 pandemic on the study or research, and the online learning during the lockdown (the effect of lockdown on academic performance, electronic device used to study online, virtual learning tools used, time spent per day in online learning, evaluation of online learning both in the theoretical or practical parts, common problems encountered in the online learning, and suggestions to improve the online learning). This section consisted of ten questions as follow: three single-choice questions, three multiple-choice questions, one Likert-scale question, and three questions with free text answer.

### Data Collection

Sample size was calculated to be 384 participants as a minimum number of participants (22). Data collection was done using a spreadsheet linked to the online google form questionnaire. Data collection was done during the period from April 13^th^ to August 5^th^ 2020.

### Statistical Analysis

Data were exported and analyzed using SPSS version 21.0 (IBM Corporation). Descriptive statistics were presented as counts and percentages to summarize the collected data. To measure the effect of COVID-19 lockdown on the academic performance of veterinary medical students, 5-Point Likert Scale was used. Answers were converted into numeric values as follow (greatly affected = 5 points; considerably affected = 4 points; moderately affected = 3 points; slightly affected = 2 points; not affected = 1 point) (23). To evaluate the online education during the pandemic lockdown, a 10-Point Likert Scale was used. Participants were asked to evaluate the online education in general, and the online education in practical lessons during the lockdown (1 was the lowest evaluation and 10 was the highest evaluation).

## Results

### Demographic Characteristics of Participants

**Figure 1:**
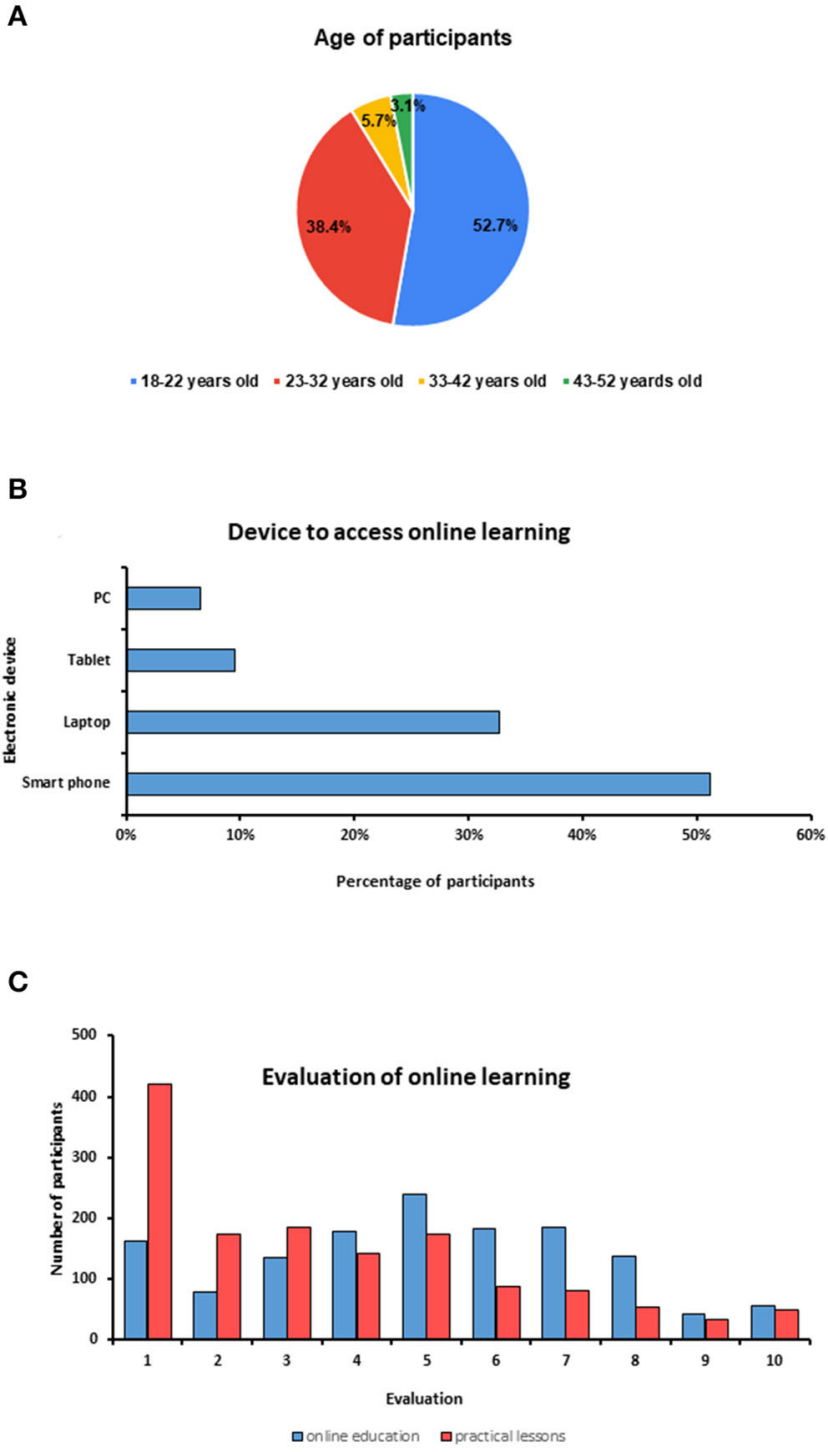
**(A)** The distribution of participants according to their age. **(B)** A diagram showing the device used by participants to access online materials. **(C)** Evaluation of online learning.

A total of 1,479 responses were retrieved of which 87 responses were excluded due to mismatched data. The remaining 1,392 responses were from 92 different countries with an overall response rate of 94.1% (Table 1). Of the 1,392 participants, 674 (48.4%) were males, and 718 (51.6%) were females (Table 2). The age of participants ranged from 18 to 52 years (mean ± SD = 24.10 ± 5.93 years). About 52.7% of the participants were aged 18–22 years, 38.5% were aged 23–32 years, 5.7% were aged 33–42 years, and 3.1% were aged 43–52 years (Figure 1A). The majority of the participants (80.7%, *n* = 1,122) were undergraduate students while postgraduate students comprised about 19.3% (*n* = 270). About 55.7% (*n* = 775) of the participants were residents in a city while 44.3% (*n* = 617) the participants were residents in rural areas (Table 2).

**Table 1:** Country list and the number of participants.

**Country**	**Number**	**Country**	**Number**	**Country**	**Number**	**Country**	**Number**
Egypt	151	Belgium	14	Lithuania	5	Canada	2
India	122	Slovakia	14	Myanmar	5	Denmark	2
Philippines	100	Tanzania	13	Palestine	5	Estonia	2
Pakistan	74	Kosovo	13	Puerto Rico	5	Guatemala	2
Nigeria	63	Iraq	11	Sri Lanka	5	Hungary	2
Australia	53	Zambia	11	South Sudan	5	Mongolia	2
Indonesia	40	Brazil	9	Sudan	5	Norway	2
Somalia	41	Perú	9	Sweden	5	Portugal	2
Kenya	41	Uganda	9	Taiwan	5	Senegal	2
Poland	39	Algeria	8	Vietnam	5	Serbia	2
USA	38	Colombia	8	Cameroon	4	Syria	2
Croatia	35	Italy	7	Laos	4	Yemen	2
UK	32	Ukraine	7	Malaysia	4	Azerbaijan	1
Romania	31	Greece	7	Moldova	4	Costa Rica	1
Jordan	28	Czech republic	6	Russia	4	Gambia	1
Thailand	26	Bosnia and Herzegovina	6	The Netherlands	4	Kazakhstan	1
Ghana	26	Ireland	6	Botswana	3	North Cyprus	1
Mexico	23	France	6	Hong Kong	3	North Macedonia	1
Nepal	23	Lebanon	6	Japan	3	Switzerland	1
South Africa	18	Libya	6	New Zealand	3	Turkey	1
Bulgaria	18	Afghanistan	5	Rwanda	3		
Ethiopia	17	Albania	5	Spain	3		
Morocco	15	Argentina	5	Austria	2		
Bangladesh	14	Germany	5	Cambodia	2		

**Table 2:** Sociodemographic characteristics of participants.

**Variables**	**Number**	**Male**	**Female**
Total number	1,392	674 (48.4%)	718 (51.6%)
**Educational level**			
Undergraduate students	1,122 (80.7%)	490	632
1^st^ year students	200 (14.4%)	99	101
2^nd^ year students	268 (19.2%)	120	148
3^rd^ year students	233 (16.7%)	103	130
4^th^ year students	195 (14.0%)	71	124
5^th^ year students	145 (10.4%)	51	94
6^th^ year students	81 (5.8%)	46	35
Postgraduate students	270 (19.3%)	184	86
Diploma	110 (7.9%)	82	28
Master students	104 (7.4%)	67	37
PhD student s	56 (4.0%)	35	21
**Residential area**			
City	775 (55.7%)		
Rural area	617 (44.3%)		

### The Effect of COVID-19 Pandemic Lockdown on Academic Performance

The current data showed that the average evaluation was 4.02 ± 1.11 points. Most of participants 96.7% (*n* = 1,346) believed that COVID-19 pandemic lockdown affected their academic performance with varying degrees. Nearly half of the participants (47.5%, *n* = 661) were greatly affected whereas 19.9% (*n* = 278) were considerably affected, 23.3% (*n* = 324) moderately affected, and 6.0% (*n* = 83) were slightly affected. Whereas, only 3.3% (*n* = 46) of participants reported that lockdown had no effect on their academic performance (Table 3).

**Table 3:** Impact of COVID-19 lockdown on participants' performance.

**Variables**	**Greatly affected (5)**	**Considerably affected (4)**	**Moderately affected (3)**	**Slightly affected (2)**	**Not affected (1)**
**Undergraduate students**					
1^st^ year students	87 (43.5%)	46 (23.0%)	51 (25.5%)	11 (5.5%)	5 (2.5%)
2^nd^ year students	126 (47.0%)	44 (16.4%)	68 (25.4%)	22 (8.2%)	8 (3.0%)
3^rd^ year students	116 (49.8%)	54 (23.2%)	49 (21.0%)	10 (4.3%)	4 (1.7%)
4^th^ year students	97 (49.7%)	43 (22.1%)	41 (21.0%)	9 (4.6%)	5 (2.6%)
5^th^ year students	77 (53.1%)	34 (23.4%)	28 (19.3%)	5 (3.5%)	1 (0.7%)
6^th^ year students	41 (50.6%)	17 (21.0%)	15 (18.5%)	5 (6.2%)	3 (3.7%)
**Postgraduate students**					
Diploma	52 (47.3%)	8 (7.2%)	33 (30.0%)	9 (8.2%)	8 (7.3%)
Master students	45 (43.2%)	19 (18.3%)	27 (26.0%)	7 (6.7%)	6 (5.8%)
PhD students	20 (35.7%)	13 (23.3%)	12 (21.4%)	5 (8.9%)	6 (10.7%)

### Evaluation of Online Education During COVID-19 Pandemic Lockdown

Data showed that participants used several electronic devices to study online. The most used device was the smartphone (51.0%) followed by laptop (32.8%) and tablet (9.6%), while the least used device was the personal computer (6.6%) (Figure 1A). The studying hours spent for online learning ranged from <1 h/day to 14 h/day with an average of 3.1 ± 1.9 h/day. Regarding the frequency of online studying hours, about 44.7% (*n* = 622) of participants spent up to 2 h/day in online learning, while 48.8% (*n* = 679) of participants spent 3–6 h/day, and 6.5% (*n* = 91) of participants spent 7–14 h/day.

The mean evaluation score for the online education in general was 5.1 ± 2.4 while that for the practical parts was 3.6 ± 2.6. About 56.9% (*n* = 792) of participants evaluated the online learning in general with 1–5 of 10 points, while 78.4% (*n* = 1,091) of participants evaluated the online learning in practical lessons with 1–5 of 10 points (Figure 1B).

**Figure 2:**
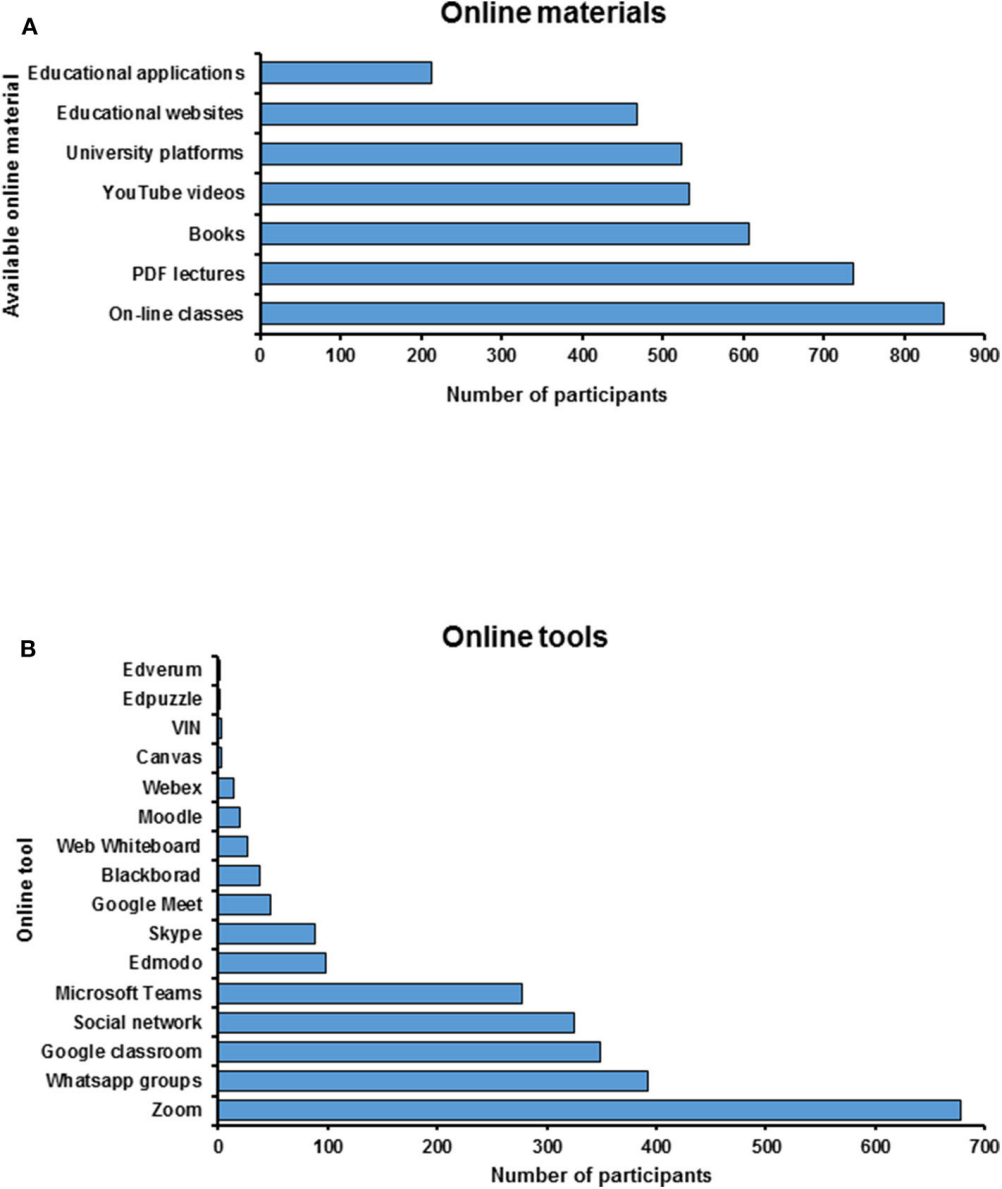
A diagram showing **(A)** the available online materials and **(B)** online tools used by participants to access the study materials online.

Participants showed that the online study materials were available mostly through online classes and pdf lectures followed by e-books, YouTube videos, university platforms, educational websites, and educational applications (Figure 2A). Different online tools had been used to access the online classes. The distribution of these online tools was as follow; Zoom had the highest preference followed by WhatsApp, Google classroom, and social networks. Microsoft Teams, Edmodo, Skype, and Google Meet were moderately used. While Canvas, Edpuzzle, Adobe connect, and Edverum were not popular tools (Figure 2B).

### Advantages of Online Learning of Veterinary Medical Sciences

The advantages of online learning according to the opinion of some students could be summarized as follow:

- More convenient and flexible than ordinary classes
- Students have more time to learn and do other activities
- Saves time and provides an opportunity for self-study

### The Common Problems With Online Learning of Veterinary Sciences

The participants' responses regarding to the common problems with online learning could be summarized as follow:

- Loss of interest
- The availability of internet to students live in provincial and rural areas.
- Speed and cost of internet hinder proper delivery of study materials by both students and lecturers.
- The availability of learning devices, such as laptops, tablets, and smartphones devices to access the internet and view the online materials.
- The shortness of the available time to solve the online tests, which causes panic.
- Lack of application in the clinical setting for the things we learned from book.
- Lack of online information about certain subjects, such as veterinary anatomy.
- It is hard to teach the practical lessons of clinical subjects in online basis.
- Spending long time in online learning makes the students loss their motivation to participate, also they feel tired with sleeping issues.
- The availability of online resources, some lectures are provided in PowerPoint or pdf format, or lecturers just read from PowerPoint slides.
- Less interactive due to no contact between students, professors and animals, which makes it very boring and easily lose concentration.
- Lack of effective communication.
- Some students have the sense of loneliness.

### Recommendations to Improve Online Learning in Veterinary Science

The students' recommendations regarding improvement of the online learning were summarized as follows:

- The universities should provide platforms for online learning with easy access to the study materials.
- Provide students with electronic devices, such as computers, and tablets to access the internet.
- Improvement of internet speed and providing cheaper or even free internet packages during the pandemic.
- Provide training for lecturers on e-learning tools and computer skills.
- Improve the way of teaching to encourage students to learn and attract them to study online.
- Provide virtual resources to mimic the laboratory work or live streaming directly from the laboratory.
- Enhance the interaction between students and teachers (for example with Mentimeter application).
- Practical learning throughout interactive tools, such as videos and 3D animation is significantly more effective than text materials such as power point and pdf, voice recordings should be provided with the lecture's text.
- Provide accessible online resources such as e-books and instructional videos for practical lessons.
- Decrease the amount of classwork could help reducing students' stress.
- Provide online quizzes and assignments after every lesson to measure the degree of students' understanding.
- Increase the available time to solve the online tests.

## Discussion

The novel COVID-19 disease identified in Wuhan city, China in December 2019 spreads rapidly not only in China, but also worldwide. Therefore, governments around the world have either temporarily closed or implemented localized closures of educational institutions affecting over 60% of student population worldwide (10). About 155 countries worldwide have introduced various tools and learning platforms as a solutions to continue the education process during the pandemic (24).

Many universities around the world have minimized gatherings through suspending or canceling all campus activities including suspension of classroom teaching to decrease the rapid spread of virus. Consequently, several colleges and universities worldwide switch to the online teaching for undergraduate and graduate students (12) to minimize either the contact either between the students and lecturers or between students themselves (8).

Our data showed that 1,392 participants from 92 countries answered the questionnaire, which represented an overall response rate of 94.1%. Participants were 48.4 and 51.6% males and females, respectively. The majority of the participants (80.7%) were undergraduate students while postgraduate students comprised 19.3%. The current data showed that COVID-19 pandemic lockdown affected the academic performance of most participants 96.7% with varying degrees. This is in agreement with previous studies, which reported that COVID-19 has a profound impact on medical students, dental medical students, and radiology trainee (15–20). Taking online courses has a negative effect on students; reduction of students' progress and success has been reported to be associated with taking online college courses, instead of traditional in-person courses (25).

The current study showed that the most popular device that students used to access the online materials was the smart phone followed by laptop, while the least used tool was the personal computer. This result is in accordance with the results reporting that students use smart phones and laptops at higher rates followed by iPads/tablets then PC to access online mathematics lessons (26) and social media (27). In this regard, Lazarus et al. (28) showed that the use of mobile devices in studying anatomy among medical students in South Africa has a positive impact on students' learning experience. It is worth to mention that many students have no access to the online teaching due to lack of either the means or the instruments because of economical and digital divide (14). Unequal access to computers and internet alters the effectiveness of online learning (29).

The studying hours spent for online learning ranged from <1 h/day to 12 h/day. Other than live streaming, students can access the online materials at any hour of the day when convenient to them. This flexibility helps some students to better invest their time and efforts while it is considered as a challenge to other students who cannot manage their own time (25). Our data showed that Zoom had the highest preference followed by WhatsApp, and Google classroom while Microsoft Teams, Edmodo, Skype, and Google Meet were moderately used in their online learning. It has been reported that freely available software, such as Zoom, Google Meet, Microsoft Teams, and WebEx are used widely in online teaching of medicine than others (30). Moreover, Malhotra and Bansal (31) reported the wide usage of WhatsApp in academic purposes by veterinary students. Undergraduate students use it to share images, educational videos, and links of educational websites while postgraduate students use it to discuss their research projects, share experimental results and research findings, and exchange of academic experiences (31).

The vast development in communication and information technology has impacted delivery and quality of education. Virtual classrooms and instructors have replaced traditional classrooms in several university courses worldwide (32). This novel way of teaching has been welcomed by majority of students due to its flexibility, convenience, and lower cost (33). Distance learning in veterinary medicine is considered as an alternative and effective way to deliver information rather than a substitute for the traditional classroom. Therefore, it is recommended to use a distance learning together with traditional teaching methods (34).

Both veterinary and medical students showed a higher motivation to the web-based learning of anatomy, morphology, and surgery than traditional teaching in both developed and developing countries (28, 33, 35–37). In addition, medical students prefer histology virtual microscope laboratory than regular microscope laboratory (38).

The most common problems associated with online education in general included the availability of internet in provincial and rural areas, the speed and cost of internet, the availability of electronic devices to access the internet, and the lack of interaction between students and lecturers. While specific problems associated with online education of subjects of veterinary science included lack of application of the clinical setting, lack of online information about certain subjects, such as veterinary anatomy, challenging of teaching the practical lessons online, and lack of contact with animals.

To improve online education in general it is recommended to provide platforms for online learning, provide students with electronic devices to access the internet, improve the internet speed, provide cheaper or even free internet packages during the pandemic, provide professional training for lecturers, and enhance the interaction between students and teachers. Additionally, to improve online education in veterinary science it is recommended to provide virtual resources to mimic the laboratory work, teach practical lessons by interactive tools, such as videos and 3D animation, and provide accessible e-books and instructional videos for practical lessons.

## Concluding Remarks

The current study showed that COVID-19 pandemic lockdown affected the academic performance of most participants with varying degrees. Online education helps to keep the students up and running with an opportunity for self-study. However, the main challenge online education faces in veterinary medical science is how to give practical lessons. Since most of the subjects are practical; therefore, it is not easy to learn it online. Students think that it is difficult to fulfill the veterinary competencies only with online education system. Online education can be improved by making it more interactive, showing medical procedures in real situations, giving concise information, and providing 3D virtual tools to mimic the real situation.

## Data Availability Statement

The data are available from the corresponding author upon reasonable request.

## Ethics Statement

The studies involving human participants were reviewed and approved by the institutional ethics committee of the South Valley University, Egypt. Participants agreed to participate in the present study upon answering of the questionnaire.

## Author Contributions

MM: designed the questionnaire, analysis of result, wrote the manuscript and approved it.

## Conflict of Interest

The author declares that the research was conducted in the absence of any commercial or financial relationships that could be construed as a potential conflict of interest.
