# Supplementary material for: The Impact of COVID-19 Pandemic on the Academic Performance of Veterinary Medical Students
Source: Front Vet Sci. 2020 Oct 6;7:594261. doi: 10.3389/fvets.2020.594261 (PMC7572855; doi:10.3389/fvets.2020.594261)
Supplement: Supplementary file 1 [file Data_Sheet_1.docx]

Supplementary materials 1: Online questionnaire

**Impact of COVID-19 and academic performance of veterinary medical students**

This questionnaire aims to analyze the effect of COVID-19 pandemic on the academic performance of veterinary medical students and researchers during the lockdown. Both undergraduate and postgraduate veterinary medical students are eligible to answer this questionnaire. The data of this questionnaire will be used for a research study and will be published in an international journal.

Your identity is anonymous, and your participation in this study is completely voluntary. Answering this questionnaire indicates your consent to participate in this study.

Dr. Mohamed Mahdy,

m_mahdi@vet.svu.edu.eg

Section A: Demographic characteristics

1. What is your gender?

- Male
- Female

2. What is your age?

……….. years old

3. What is your country?

………………………………………………….

4. Where do you live?

- City
- Rural area

5. Which university do you enroll in?

…………………………………………………...

6. What is the level of your study?

- Undergraduate veterinary student
- Postgraduate veterinary student

7. For undergraduate students: In which academic year are you?

- First academic year
- Second academic year
- Third academic year
- Fourth academic year
- Fifth academic year
- Sixth academic year

8. For postgraduate students: In which postgraduate course are you currently enroll in?

- Diploma
- Masters
- PhD

Section B: Assessing the impact of COVID-19 pandemic on the academic performance

**1. How did COVID-19 pandemic affect your study/ research?**

- Greatly affected (5)
- Considerably affected (4)
- Moderately affected (3)
- Slightly affected (2)
- Not affected (1)

**2. Which electronic device do you use to study online during lockdown?**

- Laptop
- PC
- Smart phone
- Tablet

**3. How many hours do you spend in on-line learning during lockdown?**

- 1 hour/ day
- 2 hours/ day
- 3 hours/ day
- 4 hours/ day
- 5 hours/ day
- 6 hours/ day
- 7 hours/ day
- 8 hours/ day
- 9 hours/ day
- 10 hours/ day
- Other

**4. How do you rate on-line education during COVID-19 pandemic? (1 is the lowest evaluation)**

**○ ○ ○ ○ ○ ○**  **○ ○ ○ ○**

1 2 3 4 5 6 7 8 9 10

**5. How do you rate on-line education in practical lessons during COVID-19 pandemic? (1 is the lowest evaluation)**

**○ ○ ○ ○ ○ ○**  **○ ○ ○ ○**

1 2 3 4 5 6 7 8 9 10

**6. Which virtual learning tools do you use during the lockdown?**

- University platforms
- On-line classes
- Educational websites
- YouTube videos
- E. Books
- Educational applications
- PDF lectures
- Others

**7- Which online learning tool do you use during the lockdown?**

- Zoom
- Microsoft Teams
- Skype
- Web Whiteboard
- Edmodo
- Google Meet
- Social networks
- WhatsApp
- Others

**8- What are the common problems associated with online learning during the lockdown?**

……………………………………………………………………

**9. How can we improve online education?**

……………………………………………………………………

**10. Comments and/or opinion**

………………………………………………………………………
